# Surgical Skills Workshops Should Be a Part of the United Kingdom Undergraduate Medical Curriculum

**DOI:** 10.7759/cureus.4642

**Published:** 2019-05-11

**Authors:** Muhammad A Hakim, Elizabeth D. Dominguez, Sebastian Priest, Keng Siang Lee, Ameen Mardanpour, Sankalp Tandle, Majid Al-Khalil, George Slade, Sameer Gujral

**Affiliations:** 1 Health Sciences, University of Bristol Medical School, Bristol, GBR; 2 Orthopaedics, University of Bristol Medical School, Bristol, GBR; 3 Plastic Surgery, Southmead Hospital, Bristol, GBR

**Keywords:** surgical skills, teaching, workshops, medical student curriculum, undergraduate education, united kingdom (uk)

## Abstract

Introduction: Medical students across the United Kingdom (UK) report poor satisfaction with surgical teaching. The Surgical Skills Day (SSD) begins to address this by exposing medical students to surgery through an easily accessible one-day practical workshop. This study shows how the SSD encourages undergraduate engagement in surgery.

Method: Feedback forms were emailed to attendees of the SSD and their anonymised responses were used to evaluate the SSD.

Results: A total of 144 students attended the SSD across three years and the feedback response rate was 74% (n = 107). Key findings were that 100% of respondents (n = 107) would like the SSD to be an annual event, 79% (n = 83) were more inclined to pursue a surgical career following the event, and 97% (n = 103) would like to see practical surgical skills incorporated into the curriculum. The SSD was able to engage undergraduates with surgery through mentorship, practical skills, specialty exposure, and teaching of the General Medical Council (GMC) mandated skills.

Conclusions: Undergraduate surgical teaching in the UK is insufficient. The student-led annual SSD showed improved engagement in practical surgical skills and increased enthusiasm for a surgical career. In light of this, the authors feel the SSD or similar event should be integrated into the UK medical school curriculum.

## Introduction

In 2015, the Royal College of Surgeons (RCS) of England highlighted the need for a robust undergraduate surgical education [1]. However, medical students across the United Kingdom (UK) report poor satisfaction with surgical teaching and inadequate preparation for surgical rotations during foundation training when compared to medical rotations [2-4]. Furthermore, many junior doctors lack competency in basic surgical skills, such as skin suturing, which are mandated by the General Medical Council (GMC) for all newly qualified UK doctors [5-8].

Surgical skills workshops are a promising initiative to compensate for the aforementioned lack of formal undergraduate surgical education; it not only improves medical students’ proficiency in suturing but also exposes them to surgical specialties and stimulates their interests in surgery as a career option [9-10].

The annual Surgical Skills Day (SSD), organised by the University of Bristol Surgery Society (SCRUBS), is a student-led surgical skills workshop. This article reports conclusions from three annual SSDs (2016 - 2018), based on feedback from the University of Bristol medical students.

## Materials and methods

The SSD is a one-day practical course aimed at the University of Bristol medical students. The day is comprised of a range of diverse practical surgical skill stations, each one hour in duration. The instructors running the workshop stations are surgeons of different grades from trainee to consultant level. The event was priced at five pounds (lunch inclusive) to help with equipment hire and food purchase. Across the three years of running the SSD, workshop stations have included laparoscopic simulation, dynamic hip screw placement using model femurs, burr hole drilling on model skulls, tracheostomy insertion on mannequins, trauma scenarios focusing on conducting a primary survey, porcine aortic re-anastomosis, tendon repair, and suturing using porcine models (see Table 1 below for details on stations provided each year). After completion of the SSD, attendees were asked to complete an anonymised feedback form (see Appendix) in exchange for a certificate of workshop completion, to incentivise students to provide feedback.

**Table 1:** Different Workshop Stations for 2016-2018

Year	Workshop Stations
2016	Suturing, Dynamic Hip Screw, Laparoscopic Simulation, Burr Hole Drilling, Trauma Scenarios
2017	Suturing, Dynamic Hip Screw, Laparoscopic Simulation, Tendon Repair
2018	Suturing, Laparoscopic Simulation, Tendon Repair, Aortic Re-Anastomosis, Tracheostomy

## Results

We have listed some of the key details below from the 2016 - 2018 SSD feedback responses.

Over the course of three years, an average of 48 participants (2016 - 2018 average (standard deviation (SD): 6.48)) attended the SSD each year for a total of 144 students. In 2016, 57 students attended, with 42 in 2017 and 45 in 2018. The mean response rate for feedback was 74% (n = 107) (2016 - 2018 average (SD: 10.5)). Attendance was divided 47% (n = 50) and 53% (n = 56) between males and females (2016 - 2018 average (SD: 1.83)), respectively. The percentage of students either in the first, second, or third year was an average of 64% (n = 68); the remaining 36% (n = 38) of attendees were in their fourth or fifth year of study (2016 - 2018 average (SD: 13.9)).

**Figure 1:**
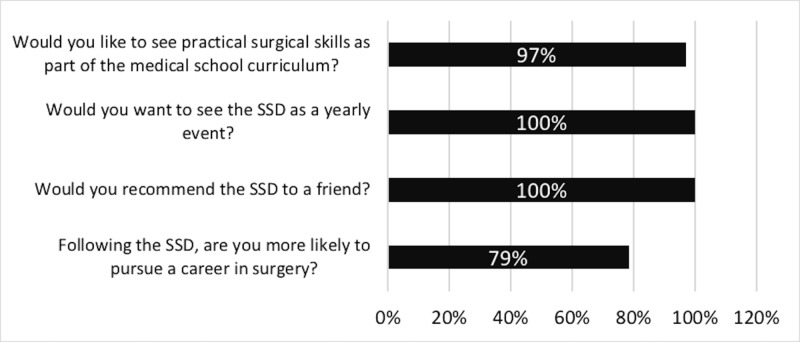
Bar graph depicting the percentage of students who answered “yes” to the following questions

Figure 1 below shows student responses across the three years to key questions of the feedback form.

**Figure 2:**
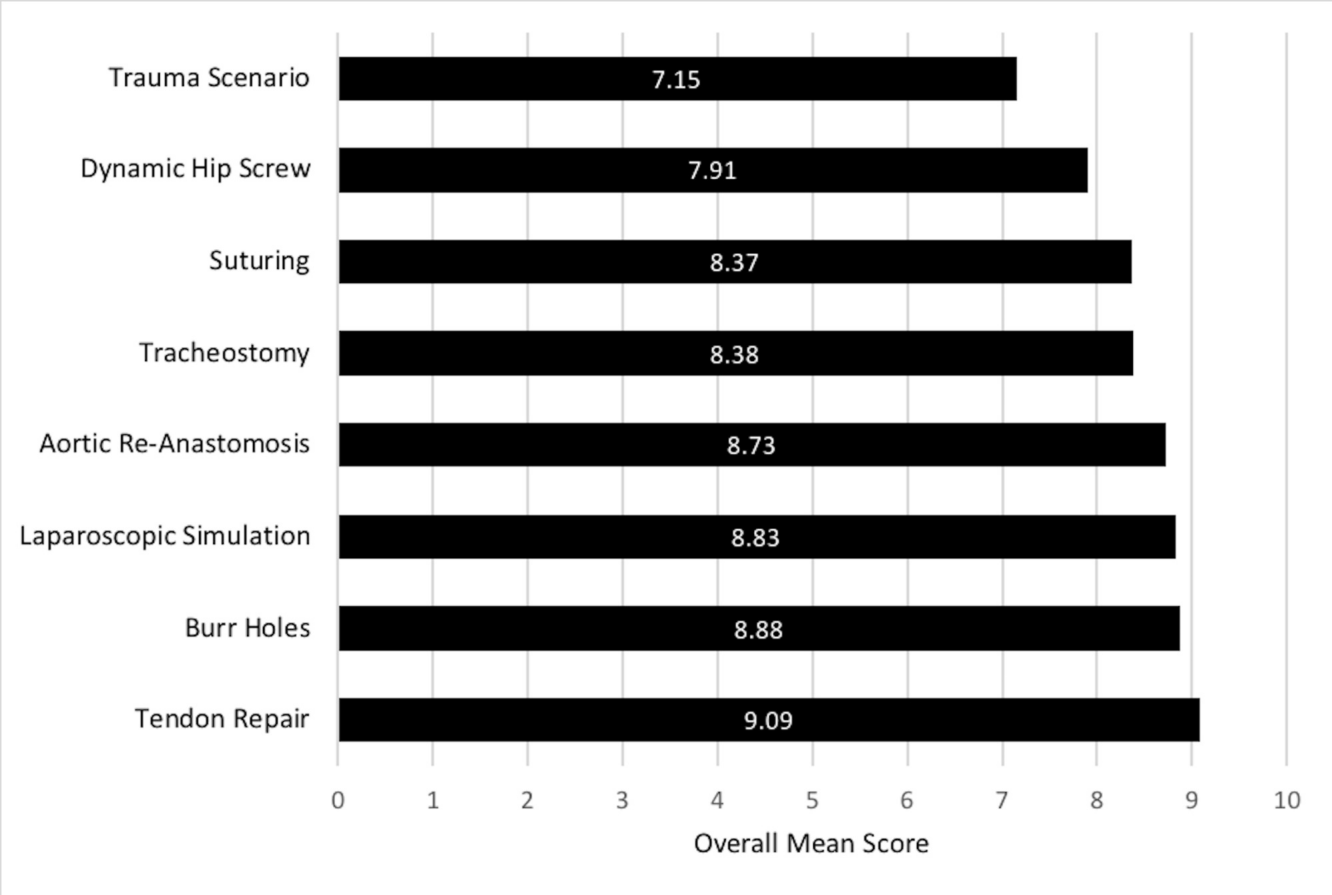
Mean rating (on a scale of 1 to 10) for overall satisfaction of each workshop station

The feedback questionnaire required attendees to score each workshop station for overall satisfaction, on a rating scale of 1 to 10, with 10 being the highest. The mean rating for each station is shown below in Figure 2.

The feedback questionnaire asked students to comment on the surgeons who were instructing the workshops. Table 2 below shows the percentage of positive and negative comments regarding instructor performance for the 2018 SSD. Feedback comments for instructor performance and interactions are detailed below in Table 3 (2016 and 2017 data cannot be provided as instructor performance was not assessed during these years).

**Table 2:** Feedback Comments on Instructors for the 2018 Surgical Skills Day

Category	Number of Responses (%)
Positive comments	48 (71.5)
Negative comments	20 (28.5)

**Table 3:** Feedback Comments for Instructors from Different Workshop Stations for the 2018 Surgical Skills Day

Quantitative analysis of comments in response to: ‘Please comment on the Suturing instructor(s). What did they do well and what could they improve on?’	
Category	Number of Responses (%)
Excellent quality of tuition	51 (47.9)
Approachable, informative, and supportive tutor	15 (14.3)
Provided the opportunity to develop previously learned skills	7 (6.30)
Positively challenging, i.e., ‘Pushing us to get better’	4 (4.20)
Total (positive comments)	78 (72.7)
Lack of/unsuitable equipment provided	7 (6.30)
Should allocate groups based on ability	7 (6.30)
Should improve guidance	7 (6.30)
More tutors should have been available	4 (4.20)
Should have featured a small theoretical introduction	2 (2.10)
Not appropriate for training level, i.e., ‘I didn’t learn anything new’	2 (2.10)
Total (negative comments)	29 (27.3)

Quantitative analysis of comments in response to: ‘Please comment on the ENT instructor(s). What did they do well and what could they improve on?’	
Category	Number of Responses (%)
Synthesis of theoretical and practical elements	12 (30.0)
Excellent quality of tuition	7 (17.5)
Approachable, informative, and supportive tutor	6 (15.0)
Excellent group size, i.e., ‘Small groups were helpful’	2 (5.00)
Tuition relevant to studies	1 (2.50)
Total (positive comments)	28 (70.0)
Should improve time management, i.e., ‘We ran out of time’	8 (2.00)
Not enough practical elements	3 (7.50)
Lack of/unsuitable equipment provided	2 (5.00)
Not appropriate for training level, i.e., ‘I didn’t learn anything new’	1 (2.50)
Total (negative comments)	12 (30.0)

Quantitative analysis of comments in response to: ‘Please comment on the Laparoscopic instructor(s). What did they do well and what could they improve on?’	
Category	Number of Responses (%)
Approachable, informative, and supportive tutor	30 (28.0)
Should improve guidance	20 (19.0)
Excellent quality of tuition	15 (14.0)
Challenging and competitive aspects provided	13 (12.1)
Tuition relevant to studies	5 (4.67)
Good range of equipment	5 (4.67)
Total (positive comments)	69 (64.5)
Should improve time management, i.e., ‘We ran out of time’	8 (7.48)
More tutors should have been available	5 (4.67)
Clinical applications not made clear	5 (4.67)
Total (negative comments)	38 (35.5)

Quantitative analysis of comments in response to: ‘Please comment on the Plastics instructor(s). What did they do well and what could they improve on?’	
Category	Number of Responses (%)
Excellent quality of tuition	15 (31.9)
Approachable, informative, and supportive tutor	13 (27.7)
Synthesis of theoretical and practical elements	3 (6.38)
Positively challenging, i.e., ‘Pushing us to get better’	2 (4.26)
Total (positive comments)	33 (70.2)
Not enough variety of skills taught	3 (6.38)
Should improve guidance	3 (6.38)
Assumed too much previous knowledge	3 (6.38)
Lack of information on specialty provided by tutors	2 (4.26)
Intensity of the tuition too high	2 (4.26)
Total (negative comments)	14 (29.8)

Quantitative analysis of comments in response to: ‘Please comment on the Vascular instructor(s). What did they do well and what could they improve on?’	
Category	Number of Responses (%)
Excellent quality of tuition	14 (35.0)
Approachable, informative, and supportive tutor	14 (35.0)
Tuition relevant to studies	3 (7.50)
Opportunity to develop previously learned skills	2 (5.00)
Total (positive comments)	32 (80.0)
Should improve guidance	3 (7.50)
Lack of/unsuitable equipment	1 (2.50)
Should allocate groups based on ability	1 (2.50)
Not appropriate for training level, i.e., ‘too much unfamiliar jargon’	1 (2.50)
Clinical applications not made clear	1 (2.50)
Should have featured a small theoretical introduction	1 (2.50)
Total (negative comments)	8 (20.0)

Table 4 below shows the student feedback comments across the three years on aspects they felt were particularly good about the SSD.

**Table 4:** Feedback Comments on Aspects of the Surgical Skills Day That Were Particularly Good

Category	Number of Responses (%)
“Hands-on” practicality	42 (38.5)
Variety of specialty stations on offer	28 (24.9)
Surgeon interaction	11 (10.7)
Overall organisation of the day	6 (5.70)
Enjoyment and fun	5 (5.00)
Fulfillment of dietary requirements	5 (5.00)
Challenges and Competition	4 (4.30)
Value for money	3 (3.60)
Good amount of time provided at each station	2 (2.10)

Table 5 below details aspects of the SSD across the three years which the students wanted to change.

**Table 5:** Feedback Comments on Aspects of the Surgical Skills Day That Students Wanted to Change

Category	Number of Responses (%)
More stations with different specialties	21 (19.6)
Increase time available at stations	17 (15.9)
Increase equipment available	11 (10.3)
Reduce time available at stations	10 (9.35)
Provide handouts with information	10 (9.35)
Personal protective equipment required	9 (8.41)
Increase the number of tutors	7 (6.54)
Increase the interactivity of certain stations	5 (4.67)
Adapt sessions to training level	5 (4.67)
Provide a wider variety of food options	5 (4.67)
Smaller groups per station	3 (2.80)
Provide a careers station	3 (2.80)
Host the event on an alternative day	1 (0.93)

Students were asked to identify a favourite station and then comment as to why they chose that particular station. These comments were coded and grouped into categories. Categories were then assigned percentage values based on the number of respondents that mentioned them in their feedback. Table 6 shows this information.

**Table 6:** Numerical Statistics and Feedback Comments for the Different Workshop Stations that Students Identified as Their Favourite Station

Plastic Surgery: Tendon Repair				
Year	Size of Cohort	Standard Deviation	Number of Votes as Favourite Station	Confidence Interval
2018	40	1.25	17	8.64 to 9.42
2017	27	0.834	14	8.88 to 9.5
Comments:				
Category				Number of Responses (%)
“Hands-on” practicality: interactivity and enjoyment, developing new skills and techniques, contact time with equipment, putting theory into practice, realism: “it felt real”				16 (51.6)
Surgeon interaction: quality of tuition, contact time with surgeons				9 (29.0)
Relevant to studies				3 (9.68)
Challenging and competitive aspects				3 (9.68)

Neurosurgery: Burr Holes				
Year	Size of Cohort	Standard Deviation	Number of Votes as Favourite Station	Confidence Interval
2016	40	1.02	13	8.56 to 9.2
Comments:				
Category				Number of Responses (%)
“Hands-on” practicality: interactivity and enjoyment, contact time with equipment, putting theory into practice, realism: “it felt real”				6 (46.2)
Challenging and competitive aspects				5 (38.5)
Surgeon interaction: contact time with surgeons				2 (15.4)

Laparoscopic Simulation				
Year	Size of Cohort	Standard Deviation	Number of Votes as Favourite Station	Confidence Interval
2018	40	1.30	10	8.50 to 9.30
2017	27	0.834	9	8.50 to 9.13
2016	40	0.947	14	8.49 to 9.07
Comments:				
Category				Number of Responses (%)
“Hands-on” practicality: interactivity and enjoyment, developing new skills and techniques, contact time with equipment, putting theory into practice, realism: “it felt real”				22 (66.7)
Challenging and competitive aspects				6 (18.2)
Surgeon interaction: quality of tuition, contact time with surgeons				4 (12.1)
Variety of activities on offer				1 (3.03)

Vascular Surgery: Aortic Re-Anastomosis				
Year	Size of Cohort	Standard Deviation	Number of Votes as Favourite Station	Confidence Interval
2018	40	1.43	6	8.29 to 9.17
Comments:				
Category				Number of Responses (%)
“Hands-on” practicality: interactivity and enjoyment, developing new skills and techniques, time available to develop skills				3 (50.0)
Challenging and competitive aspects				2 (33.3)
Surgeon interaction: quality of tuition				1 (16.7)

ENT: Tracheostomy				
Year	Size of Cohort	Standard Deviation	Number of Votes as Favourite Station	Confidence Interval
2018	40	1.46	5	7.93 to 8.83
Comments:				
Category				Number of Responses (%)
“Hands-on” practicality: putting theory into practice				2 (40.0)
Relevant to studies				2 (40.0)
Surgeon interaction: quality of tuition				1 (20.0)

Suturing				
Year	Size of Cohort	Standard Deviation	Number of Votes as Favourite Station	Confidence Interval
2018	40	1.12	6	8.33 to 9.03
2017	27	1.07	3	7.93 to 8.73
2016	40	1.93	7	7.48 to 8.68
Comments:				
Category:				Number of Responses (%)
“Hands-on” practicality: interactivity, time available to develop surgical skills with live tissue				7 (43.8)
Perception of learning useful skills for the future				5 (31.3)
Surgeon interaction: quality of tuition				3 (18.8)
Relevant to studies				1 (6.25)

Orthopaedic Surgery: Dynamic Hip Screw				
Year	Size of Cohort	Standard Deviation	Number of Votes as Favourite Station	Confidence Interval
2017	27	1.58	2	6.66 to 7.86
2016	40	1.05	5	8.03 to 8.67
Comments:				
Category				Number of Response (%)
Triggering personal interest in this surgical field				3 (42.9)
“Hands-on” practicality: interactivity and enjoyment, putting theory into practice				2 (28.6)
Surgeon interaction: quality of tuition				1 (14.3)
Relevant to studies				1 (14.3)

Trauma Scenario: Conducting a Primary Survey				
Year	Size of Cohort	Standard Deviation	Number of Votes as Favourite Station	Confidence Interval
2016	40	1.69	2	6.63 to 7.67
Comments:				
Category				Number of Responses (%)
Triggering personal interest in this surgical field				1 (50.0)
Perception of learning useful skills for the future				1 (50.0)

## Discussion

The SSD aims to improve surgical education at medical school and engage undergraduates in surgery at an early stage of their career. This builds enthusiasm for the profession and encourages students to make better-informed career choices. 

The SSD provides an informal environment for students to network with surgeons and develops mentor-mentee relationships. Mentorship in surgery is crucial for career development and the benefits of developing such relationships are two-way: mentorship provides personal and career enrichment to the mentee and provides satisfaction and further opportunities for the mentor [11]. Unfortunately, many students view experiences with surgeons as intimidating which is a major barrier to student engagement with the profession [12-13]. Meeting surgeons in a non-clinical setting led by students, such as the SSD, helps reduce levels of intimidation. Student comments (71.5%) reported positive interactions with the surgeons at the 2018 event (Table 2).

There is cogent evidence describing the benefits of kinaesthesia in optimising learning experiences [14]. The SSD provides kinaesthetic-style learning with "hands-on" practicality, which was deemed the most valuable aspect of the event by 38.5% of students (Table 4). Furthermore, the three highest ranked stations were tendon repair, burr hole drilling, and laparoscopic simulation (Figure 2), selected due to tactile learning opportunities; “hands-on” practicality made up 54.8% of student comments, as preferential reasons for these three stations (mean of 51.6%, 46.2%, and 66.7% for tendon repair, burr hole drilling, and laparoscopic simulation, respectively) (Table 6). 

These findings corroborate with previous studies suggesting that personal contact with surgeons, in addition to the acquisition of practical skills, is an important part of learning for undergraduates [15-16]. These learning aspects are the key components of the SSD.

The SSD exposes undergraduates to a variety of practical stations in different surgical specialties. The most valuable aspect of the SSD was reported by 24.9% of the students to be the variety of specialty exposure (Table 4). This exposure facilitates students in identifying a specialty of interest. For example, 50% and 42.9% of students reported trauma scenarios and dynamic hip screw as their favourite stations, respectively, due to interest being triggered in the specialty (Table 6). The value of exposing medical students to skills relevant to a particular surgical specialty has been reported in the literature in the context of increasing student engagement in cardiothoracic surgery [17]. In developing a surgical specialty interest, students are more likely to consider a career in surgery. This is evident in our study, with 79% of students more inclined to pursue a surgical career following the SSD (Figure 1).

In addition, the SSD provides teaching on skin suturing, an interventional procedure mandated by the GMC for all UK graduating medical students [8]. Unfortunately, the UK medical school curriculum leaves many new doctors with a lack of confidence in performing basic suturing techniques [16]. A UK national survey reported 86.5% of students received inadequate suturing training at medical school, with 21.9% feeling obliged to pay for additional surgical skills workshops [18]. The SSD addresses this issue by providing a skin suturing station in an organised workshop, teaching students GMC mandated suturing techniques. Students see the value of learning this skill, as 31.3% of attendees who identified suturing as their favourite station did so because they felt it is a useful skill for future work (Table 6).

In light of the SSD improving undergraduate engagement in surgery through mentorship, practical skills, specialty exposure, and teaching of GMC mandated skills, SCRUBS suggests an analogous event be implemented into the UK medical school curriculum. This is supported by national reviews on the medical school curriculum, as well as our attendees, 100% of whom would like the SSD to be an annual event and 97% would like to see practical surgical skills incorporated into the curriculum as shown by Figure 1 [3, 16].

## Conclusions

Surgical teaching provided to undergraduate medical students in the UK is insufficient, with regards to providing experience of practical surgical skills and teaching of GMC mandatory suturing techniques. The SSD is a student-led initiative to address these deficiencies, providing greater specialty exposure in a welcoming environment for students and surgeons to meet. In light of this, the authors suggest the SSD or similar event be integrated into the UK medical school curriculum.

## Figures and Tables

**Figure 3:**
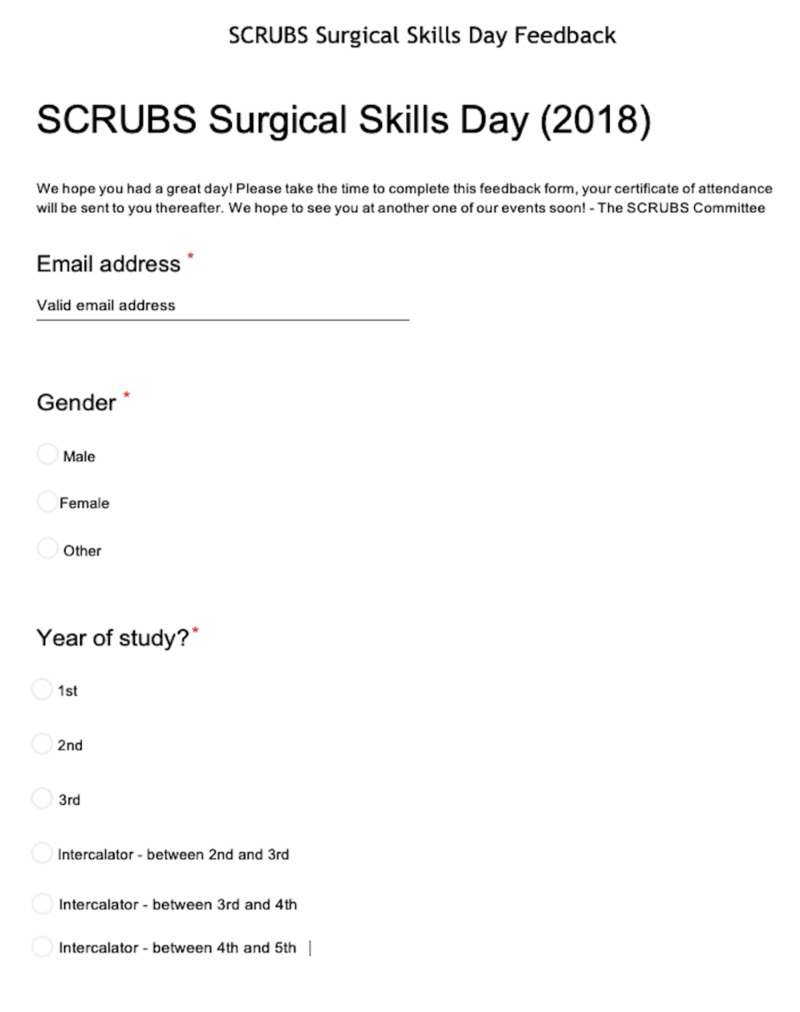
Feedback form - page 1

**Figure 4:**
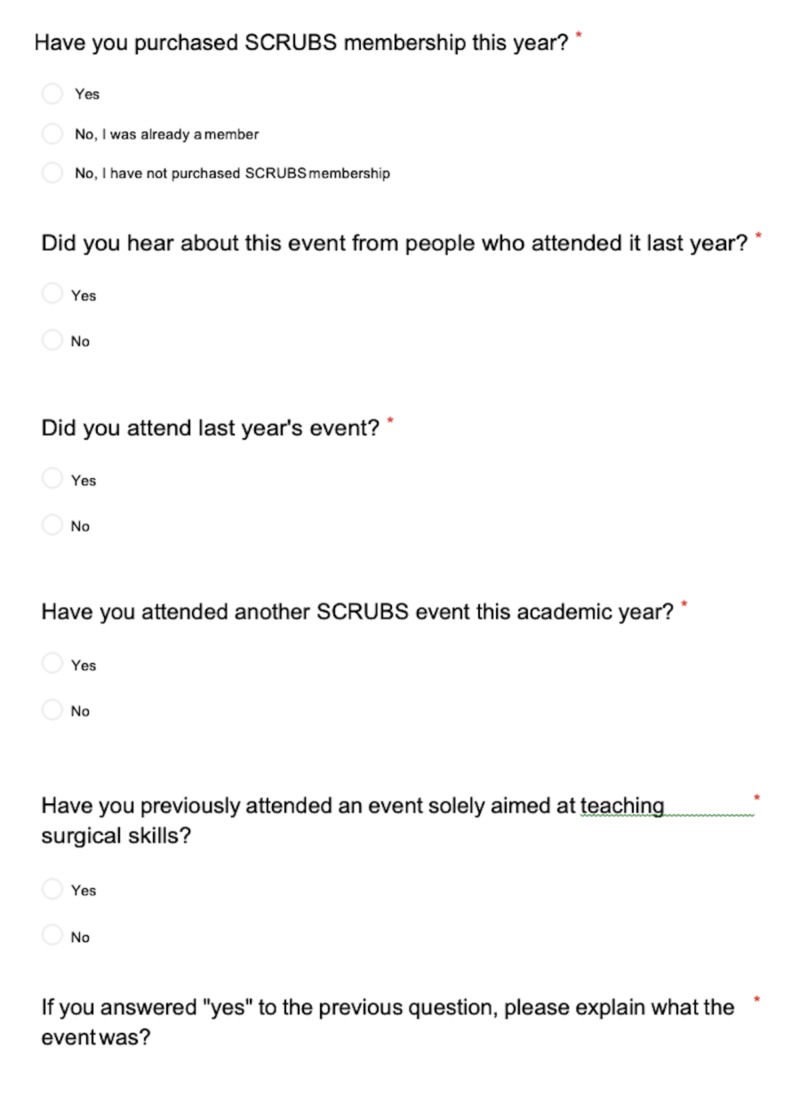
Feedback form - page 2

**Figure 5:**
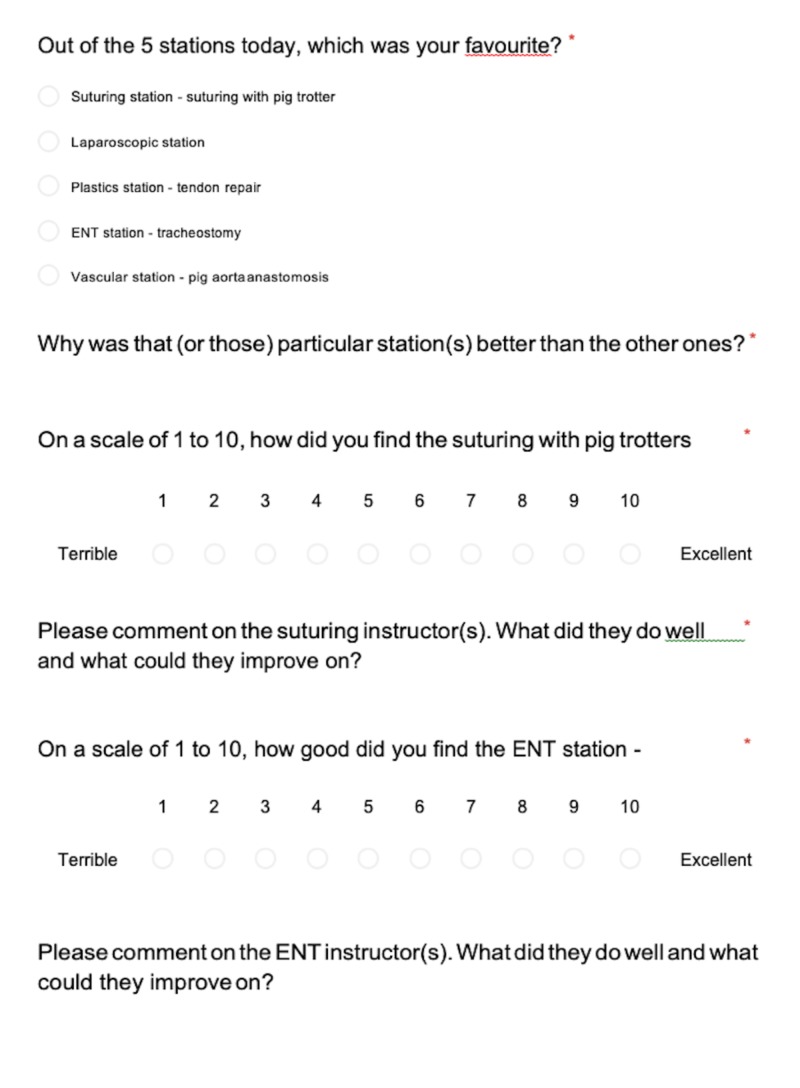
Feedback form - page 3

**Figure 6:**
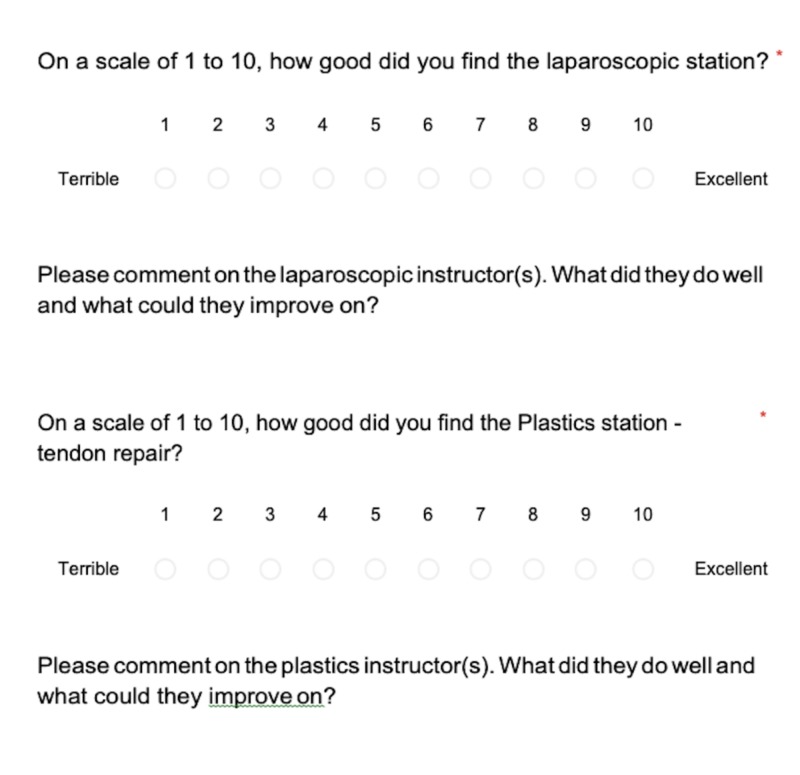
Feedback form - page 4

**Figure 7:**
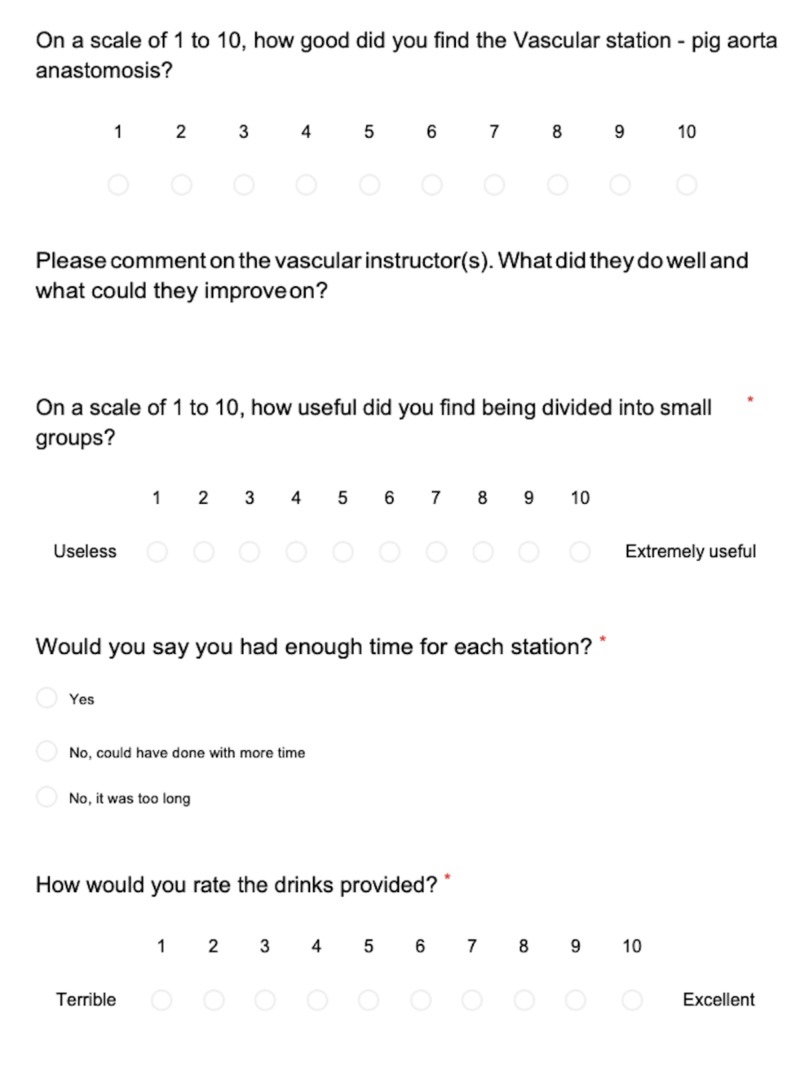
Feedback form - page 5

**Figure 8:**
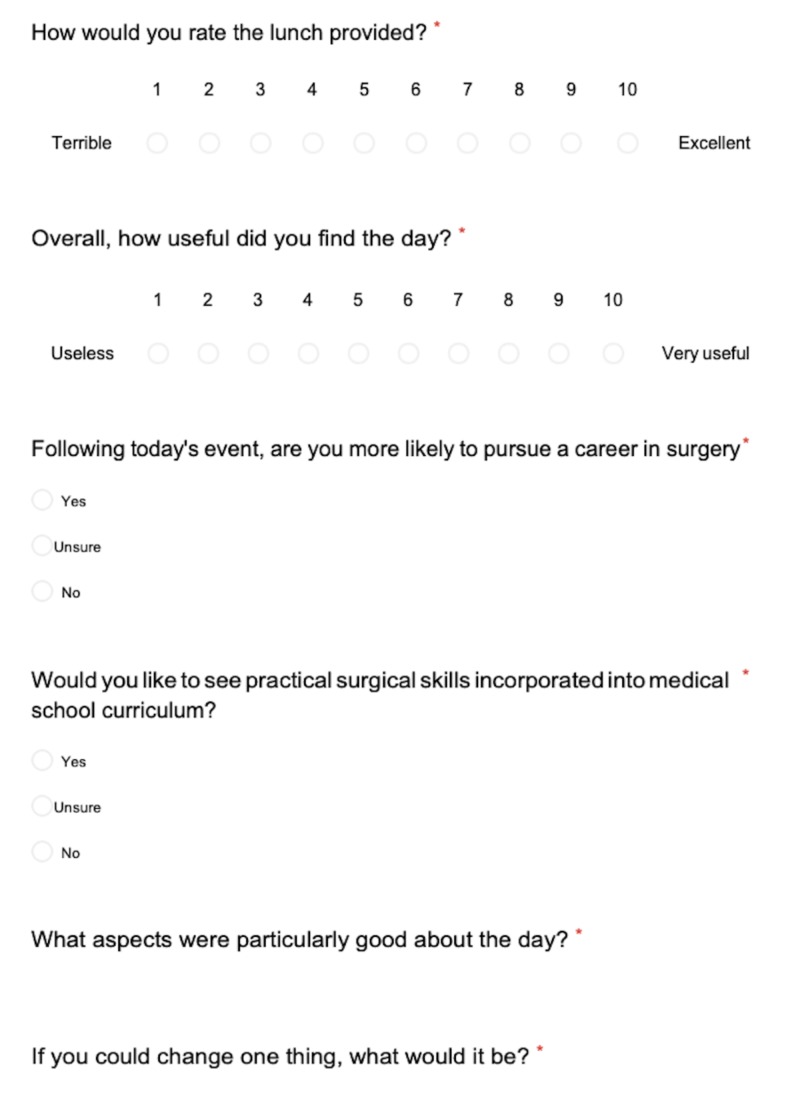
Feedback form - page 6

**Figure 9:**
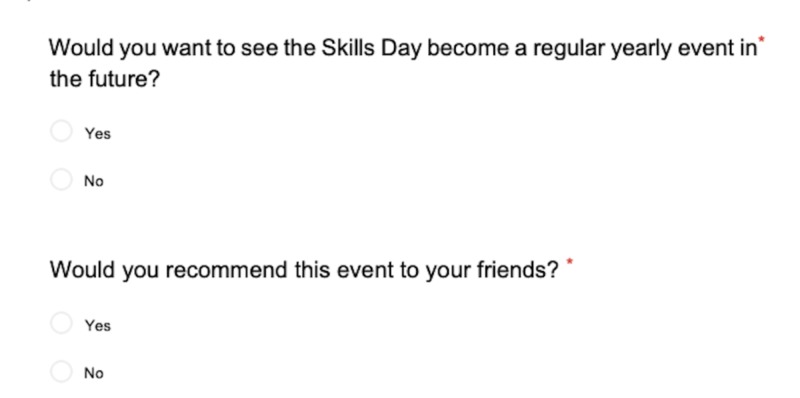
Feedback form - page 7
